# Standards for vision science librarians: 2026 review and revisions

**DOI:** 10.5195/jmla.2026.2270

**Published:** 2026-07-01

**Authors:** Heather Edmonds, Karen S. Alcorn, Caroline Allen, Rudy Barreras, Dede Rios, Leslie Holland, Thandavarayan Kumaragurupari

**Affiliations:** 1 edmondsh@neco.edu, Director of Library Services, Library, New England College of Optometry, Boston, MA; 2 karen.alcorn@mcphs.edu, Reference and Instruction Librarian and Associate Professor, Library and Learning Resources, Massachusetts College of Pharmacy and Health Sciences, Worcester, MA; 3 caroline-allen@uiowa.edu, Health Sciences Librarian, Hardin Library for the Health Sciences, University of Iowa, Iowa City, IA; 4 rbarreras@westernu.edu, Assessment and Public Relations Librarian, Harriet K. and Philip Pumerantz Library, Western University of Health Sciences, Pomona, CA; 5 dmrios1@uiwtx.edu, Director of Public Services and Community Health, Nursing, and Mexico Campuses Liaison Librarian, UIW Libraries, University of the Incarnate Word, San Antonio, TX; 6 lholland@sco.edu, Director of Library Services, Learning Resource Center, Southern College of Optometry, Memphis, TN; 7 aeh_library@aravind.org, Chief Librarian, Aravind Eye Hospital and Postgraduate Institute of Ophthalmology, Madurai, India

**Keywords:** Vision Sciences, optometry, ophthalmology, Association of Vision Science Librarians (AVSL), Standards, Professional Competence, Professional Core Competency, Collection Development

## Abstract

The Association of Vision Science Librarians (AVSL) is an international organization composed of professional librarians, or persons acting in that capacity, whose collections include vision-related materials and/or whose patrons include vision scientists. Since 1976, AVSL has defined standards for vision science libraries in all capacities, including optometry, ophthalmology, academia, industry, and beyond.

As the most recent Standards for Vision Science Libraries was published in 2014, an AVSL member committee was formed in 2024 to review the Standards and consider what a revision should include. Over the course of several months, committee members collaborated with one another, held discussions with other AVSL members, and reviewed current literature. What resulted is a revised set of Standards that aims to address the significant changes that have taken place not only within the field of librarianship, but with the information needs of the vision science professions as well.

These updated Standards place the focus on the librarian rather than the library, as many librarian positions that once focused solely on vision science are now expected to manage several disciplines. The aim of this revision is to serve as a starting point for any librarian working with vision science materials and/or serving vision scientist patrons who wishes to develop their knowledge base and skills in this particular field of health sciences librarianship.

## INTRODUCTION

The Association for Vision Science Librarians (AVSL) is an international organization composed of professional librarians, or persons acting in that capacity, whose collections include vision-related materials and whose patrons are in the vision science field [1]. AVSL members have worked collaboratively to define standards for member librarians and their respective libraries since 1976 [2]. In the last decade or so, many libraries that once had a uniquely vision-science focus have been absorbed into main campus libraries. It is not uncommon for librarian positions that once would have been entirely within the vision science domain to evolve instead into liaison roles, in which incumbents are often assigned a number of other health sciences disciplines in addition to vision science [3].

With these shifts in mind, these updates to the Standards are centered less on the library itself and more on the person who is assigned the role of vision science librarian or liaison. This role may be in any type of library: university, standalone optometric college, hospital ophthalmology department, pharmaceutical or optical corporation, professional organization, medical practice, or elsewhere. There are common expectations and considerations for librarians serving patrons who specialize in the vision sciences, regardless of locale. These revisions are based on the authors’ extensive collective experience serving these patrons, numerous conversations with colleagues serving similar patrons across all types of institutions and informed by needs and requests made by vision scientists of every type over the past several years.

The sections herein detail the knowledge base and skill set that should be developed, as well as professional standards to which the librarian responsible for vision science information services and collections should adhere. It is written primarily for librarians new to the vision science focus, but may be applicable to veteran vision science librarians, those with other health science liaison roles, and librarians new to the medical field in general. It is not the goal of these standards to instruct the new librarian on the basics; rather, the intent is for the reader to develop an understanding of what is required to become a confident librarian who is able to successfully meet the needs of patrons who work within the field of vision science.

### Qualifications

Staffing requirements for libraries that serve patrons who work in the vision sciences will vary considerably by type and size of library. Regardless of type or size, the primary staff member responsible for vision science collections and services should be a full-time, professional librarian who holds a master’s degree from an American Library Association (ALA) accredited program (or an equivalent outside of the United States).

### Scholarly Communication

Assistance with scholarly communication is a necessary service provided by the vision science librarian. The librarian should assist patrons in developing common skills needed to conduct research, such as how to cite sources properly, avoid accidental plagiarism, and use reference generators responsibly [4]. It is the librarian’s responsibility to help vision scientists understand and adhere to whatever aspect of copyright law is applicable in their respective situations: from fair use guidelines in instruction, to navigating complex reuse rights for images in published work, and beyond [5]. While librarians are not, and should not substitute for, attorneys specializing in intellectual property, the librarian will often be the first source consulted by patrons with questions about responsibly reusing existing information.

Avoiding predatory practices within publications and conferences is an ongoing concern in academia [6,7] and the vision science field is not exempt: the authors regularly field questions from patrons about the legitimacy of the solicitations they receive to publish or present. Librarians serving patrons within the vision sciences should proactively promote awareness of predatory practices, especially to early-career researchers, residents, and students. To mitigate these predatory practices within the field, AVSL established and regularly updates the Vetted List of Vision Science Journals [8] (discussed later in this article). Recognized by the Medical Library Association in 2020 with the Louise Darling Medal for Distinguished Achievement in Collection Development in the Health Sciences, the Vetted Journals List is a recommended starting point for librarians advising patrons on where to publish.

In addition to providing advice to researchers regarding the most appropriate places to submit their work for publication, librarians should offer guidance on authors’ rights. Some vision science librarians report that they maintain an institutional repository for retention and preservation of research work, and others provide data services to researchers as well. Librarians should be able to assist vision science researchers in creating and maintaining profiles across both the general science (ORCiD, etc.) and vision science scholarly communication landscape. Working with researchers to establish profiles within professional networks such as the American Academy of Optometry, the Association of Research in Vision and Ophthalmology, the American Ophthalmological Society, etc. can help to develop a wider audience for their work, connect with potential research partners, and increase the reach of their research in general. Likewise, vision science librarians should be prepared to assist researchers in evaluating and understanding their research impact through bibliometric analysis of both standard research metrics (e.g., h-index) and altmetrics.

### Literature Searching

Vision science librarians should know the most efficient and effective ways to search several different databases (all of which have their own respective search syntax and vocabularies) including but not limited to: different publishers’ versions of MEDLINE, including PubMed; Web of Science; Scopus; Cochrane Library; etc. An understanding of vision-science-related Medical Subject Headings (MeSH) is also advised. AVSL members have, for many years, annually monitored MeSH updates for any relevant changes and/or additions to vision science headings and subheadings, as well as for subjects tangential to vision science. These have been collated on the AVSL website [9].

While not every vision science librarian will be expected to participate in systematic reviews or meta-analyses, vision science researchers conducting methodological reviews of the literature should involve an expert librarian searcher in these processes [10,11]. The American Academy of Optometry’s collaboration with Cochrane Eyes and Vision to undertake a series of systematic reviews in vision science topics reflects the importance of evidence syntheses within this field [12]. Vision science librarians are encouraged to learn more about the methodological reviews undertaken by the researchers they serve, as well as obtain the appropriate level of training in the systematic review process to best support the work.

### Information Literacy and Instruction

Vision science librarians should offer learning opportunities to the communities they serve that align with the needs and level of each user group; delivery of this instructional programming should be timed and scaffolded appropriately [13]. Ideally, an information literacy framework should be developed for the vision-science-specific educational program (whether optometry, ophthalmology, PhD or MS in vision science, etc.) that both supports the specific information needs of students as they progress through the program, and develops information literacy skills that they will rely on as future researchers, educators, or practitioners. Such a framework should be mapped to institutional curricula and graduation or residency expectations, the ACRL Information Literacy Framework [14], regional information literacy accreditation standards, professional program information literacy standards (including, as of the time of writing, those in ACOE’s Professional Optometric Degree Standards [15] as well as ACGME’s Program Requirements for Graduate Medical Education in Ophthalmology [16]) and other relevant existing recommendations, such as the Association of Schools and Colleges of Optometry’s Educational Technology Guidelines [17]. Several of these are gathered in Appendix 1.

Though many patrons served by vision science librarians are researchers and/or educators, many of the abovementioned guidelines directly address the need for optometrists and ophthalmologists to understand, appraise, and apply evidence to inform clinical decisions in patient care. Evidence-based practice should, therefore, be a particular focus of the vision science librarian’s instruction, with the goal that the student, resident, or clinician understands that the highest quality eye care is informed by current best evidence in vision science [18,19].

Another contemporary consideration for vision science librarians is ensuring patrons understand the challenges and opportunities that come from the advent of artificial intelligence (AI). AI is complex, and has an impact on ethical, data privacy, and equitability issues within libraries [20]. The implications of generative AI and the proliferation of other AI-based applications on both vision science education and optometric/ophthalmic practice are also currently being explored and debated [21,22]. Clearly, there is still much to be understood about the effect of information generated by AI in research, the classroom, and the optometric or ophthalmologic practice. Vision science librarians must become AI literate in order to be able to advise patrons on its responsible and effective use in the aforementioned scenarios. While the AI landscape is ever shifting, there are some AI literacy frameworks that may be a good starting point [23,24], and librarians serving clinicians and clinical programs should give particular attention to AI literacy as pertains to patient care. Vision science librarians are presently collaborating on a nation-wide, multi-cohort research project on AI literacy in higher education to ensure the specific needs of optometrists, and health care practitioners in similar fields, are taken into account [25].

There are as many means by which instruction can be delivered as there are types of vision science patrons who require information literacy training. Instruction and programming should be tailored to the specific needs and program standards for each, whether staff or executives, clinicians, medical students, optometry students, PhD candidates, residents, fellows, or didactic faculty [26,27].

Instruction sessions are often conducted as one-shot classes taught within a library space or within a classroom, though some librarians may continuously work with a class throughout an entire semester. Hospital librarians may teach at morning rounds. Sessions should be conducted during standard working hours, but librarians may need to conduct sessions in the evenings or on weekends so that individuals with busy clinical or teaching schedules can attend. When possible, librarians should make recordings of instruction sessions available, and/or create asynchronous training materials. In hospital settings in particular, vision science librarians may be expected to perform one-on-one instruction.

### Collection Development

In developing a collection that meets the needs of the vision science community being served, there are various factors that should be taken into account, including but not restricted to resource format and accessibility, depth and extent of content, and budget limitations. To this end, the librarian should develop and/or maintain a collection development policy specifically for vision science and related resources. Having a formal policy in place can assist in justifying the inclusion or omission of requested materials. The policy should address all resource formats, including books, journals, images, multimedia, and digital content. While this may seem contrary to the prevailing wisdom on negotiating big-deal licenses and pursuing transformative agreements, the specificity of the field necessitates a more granular consideration of collection needs. This is particularly important for librarians within vision-science-specific institutions, as it is not a responsible use of materials funds to purchase packages and bundles that include content outside of the field and tangential subjects.

Vision science librarians can expect to employ their skills in critically evaluating potential additions to a collection for currency, relevancy, and authority. For those new to developing or maintaining a vision science resource collection, the following are recommended starting points for understanding the subspecialties within the field (in addition to communicating with patrons directly about their needs).

#### Classification Tables

To become familiar with the terminology and subjects within the field of vision science, the major classifications for the Library of Congress (LOC) and National Library of Medicine (NLM) can be a good starting place. Tables 1 and 2 below provide lists of classifications that encompass aspects of vision science. The vision science librarian should strive to include at least some relevant titles from each classification listed, with more or fewer in any given area depending on their patrons’ specific focuses within the field. It is recommended that these areas are collected at Level 3 or higher as defined by LOC [28].

**Table 1:** Library of Congress (LOC) Classifications Covering Aspects of Vision Science [29]

QC350-467	Optics. Light
QC450-467	Spectroscopy
QM	Human Anatomy
QP351-495	Neurophysiology and Neuropsychology
RA	Public aspects of medicine
RC321-571	Neurosciences. Biological psychiatry. Neuropsychiatry
RC346-429	Neurology. Diseases of the nervous system
RE	Ophthalmology
RJ	Pediatrics
RM	Therapeutics. Pharmacology

**Table 2:** National Library of Medicine (NLM) Classifications Covering Aspects of Vision Science [30]

QS	Human Anatomy
W	General Medicine, health professions
W18	Education
W84	Health services, delivery of health care
W87	Professional practice
W273	Vision insurance
WA	Public Health
WB	Practice of Medicine
WB18	Education
WB18.2	Educational materials
WB50	Medical practice
WB102	Clinical medicine
WB102.5	Evidence-based practice
WB290	Medical history taking
WB291	Personal health records
WB293	Collections of clinical case reports
WL	Nervous System
WL141.5	Specific diagnostic methods, A-Z
WL340	Neurologic manifestations (General, or not elsewhere classified)
WW	Ophthalmology

#### Vetted Journals List

The Vetted List of Vision Science Journals (or Vetted Journals List for short) [8] was created and is maintained by AVSL. The Vetted Journals List includes journals that have been thoroughly vetted by AVSL members using criteria informed by several sources, including OASPA’s Principles of Transparency and Best Practice in Scholarly Publishing [31]. All included journals meet high standards, and the list is updated and reviewed regularly. Prior to adding a journal to a library collection, it is recommended that the vision science librarian determine whether it has been included on the Vetted Journals List.

#### Opening Day List and Doody’s Core Titles

AVSL regularly updates the Opening Day List, a compilation of recommended books and journals that should constitute the basics of a vision science library collection in any type of institution. The Opening Day List is informed by “reserve lists, book reviews, expert opinions, faculty recommendations, usage statistics” and other pertinent criteria [32]. Vision science librarians also routinely participate in selecting for the proprietary medical collection development tool *Doody’s Core Titles*’ optometry and ophthalmology specialties, which may also be useful for the new vision science librarian looking to build a robust collection [33].

#### Recommended Databases

As previously mentioned, any library serving the needs of patrons in the vision sciences should offer, at bare minimum, access to PubMed, either Web of Science or Scopus, and (if possible) Cochrane Library for Systematic Reviews and Meta-analyses. Other databases and/or evidence-based clinical reference tools, such as DynaMed, ClinicalKey, or UpToDate, may be considered depending on the primary patron group(s) being served. Vision science librarians at institutions with an optometric focus are encouraged to subscribe to the Illinois College of Optometry’s VisionCite, which also indexes vision-specific trade publications that are not included in the aforementioned databases.

#### Image Collections

The field of vision science is highly dependent on images, primarily of the human eye, to understand and diagnose diseases and disorders. Librarians at teaching institutions should consider subscribing to robust anatomical modeling software such as Visible Body, Complete Anatomy, or the like. Acland’s Video Atlas of Human Anatomy Volume 4: Head and Neck is another suggested resource, if budget allows, as are the many print- and eBook-based subspecialty atlases available. All vision science librarians should point patrons to high-quality, freely-available online image databases curated and collated by vision scientists and professional organizations, including (at the time of writing) comprehensive collections such as the University of Iowa’s Eyerounds and University of Michigan Kellogg Eye Center’s The Eyes Have It.

#### Interlibrary Loan and Demand-Driven Access

No one library is able to own or subscribe to all necessary content, and vision science librarians must frequently fill collection gaps by utilizing interlibrary loan (ILL), cooperative borrowing agreements, and demand-driven access. AVSL facilitates resource sharing in two primary ways: via the AVSL free-reciprocal borrowing group within the National Library of Medicine’s request routing system DOCLINE [34]; and the Union List of Vision-Related Serials, a longstanding, comprehensive compilation of all current and past vision science journals that includes participating members’ holdings. While core resources should always be licensed or purchased by librarians for their patrons’ use, these shared borrowing cooperatives are crucial to ensuring access to tangential content.

When aiming to provide access to materials in related subjects, librarians at vision science institutions may find the demand-driven access eBook model economical for collection development. A burgeoning model for facilitating access to individual articles and book chapters outside of subscriptions, especially when the interlibrary loan CONTU Guidelines “Rule of 5” [35] has been exhausted, is purchase-on-demand. For libraries that are specific to vision science, canceling minimally-used subscriptions to journals in important - but peripheral - fields in favor of a purchase-on-demand model, such as that offered by Article Galaxy Scholar, has the potential to realize a significant cost savings over time. This model can typically be implemented as mediated or unmediated via the library’s link resolver.

### Networking and Collaboration

Vision science librarians should engage with the Association of Vision Science Librarians (AVSL), a free professional organization that connects librarians across the globe. Open to vision science librarians serving patrons at any type of institution, AVSL meets at least once a year in person during the Association of Schools and Colleges of Optometry (ASCO) Annual Meeting. The meeting typically includes networking with colleagues, member presentations, subcommittee reports, and a combination of both in-person and virtual components to increase participation for international members (as well as those who are not able to travel for professional development purposes). Subcommittee work continues throughout the year. Visit the Association of Vision Science Librarians website to learn more [36].

Vision science librarians can also participate in the Vision Science Caucus (VSC) of the Medical Library Association (MLA). MLA has various membership options for individuals depending on life stage and financial circumstances. There are also institutional memberships available depending on the number of full-time employees that “directly serve medical and health sciences patrons, exclusive of student, temporary or grant-funded positions [37].” VSC is free to join for MLA members, and meets at least annually either virtually or in-person.

## CONCLUSION

While vision science libraries have undergone a number of changes since the previous set of standards was published, the importance of librarians in supporting the education of vision scientists and high-quality vision research has remained constant. By focusing on continuing education, networking, and staying informed of new technologies and trends, vision science librarians can ride the wave of change that is sure to continue, confidently and competently adapting their collections and services to fulfill their patrons’ current needs.
