# Supplementary material for: Standards for vision science librarians: 2026 review and revisions
Source: J Med Libr Assoc. 2026 Jul 14;114(3):323–9. doi: 10.5195/jmla.2026.2270 (PMC13367308; doi:10.5195/jmla.2026.2270)
Supplement: Supplementary file 1 — Appendix A [file jmla-114-3-323-s01.docx]

Appendix A

[**ACOE’s Professional Optometric Degree Standards 2.19; 7.3**](https://theacoe.org/resources/professional-optometric-degree-programs) [15]

*2.19* By the time of graduation, students must be able to demonstrate an understanding of research principles and conduct to critically assess the literature.

INTENT: Certain core competencies must be attained by graduates of all accredited programs.

Examples of Evidence:

- Applicable course syllabi/course learning objectives

*7.3* The program must provide access to library services sufficient in breadth of holdings and professional staffing to support the program’s mission, goals, and objectives.

Note: When alternative modes of delivery of the educational curriculum are employed (e.g., distance education versus in-person), the program must demonstrate that provisions are in place to accommodate the needs of all stakeholders.

INTENT: The program ensures its constituents have access to library services and support needed for the fulfillment of the program’s mission, goals, and objectives.

Examples of Evidence:

- List of library holdings, including electronic resources
- Hours of library service/availability of library staff
- Organizational chart for library services
- Curricula vitae for key library personnel
- Surveys of patron satisfaction and use
- Budget allocation for library services, staff, and resources

[**ACGME Program Requirements for Graduate Medical Education in Ophthalmology 1.9.; 4.7.; 4.7.f.; 4.13.b.**](https://www.acgme.org/globalassets/pfassets/programrequirements/2025-reformatted-requirements/240_ophthalmalogy_2025_reformatted.pdf) [16]

*1.9.* Residents must have ready access to specialty-specific and other appropriate reference material in print or electronic format. This must include access to electronic medical literature databases with full text capabilities. ^(Core)^

*4.7.* ACGME Competencies – Practice-Based Learning and Improvement

Residents must demonstrate the ability to investigate and evaluate their care of patients, to appraise and assimilate scientific evidence, and to continuously improve patient care based on constant self-evaluation and lifelong learning. ^(Core)^

*4.7.f.* Residents must demonstrate competence in locating, appraising, and assimilating evidence from scientific studies related to their patients’ health problems. ^(Core)^

*4.13.b.* Residents must demonstrate competence in locating, appraising, and assimilating evidence from scientific studies related to their patients’ health problems. ^(Core)^

[**Association of Schools and Colleges of Optometry’s Educational Technology Guidelines IX. Access to Information A.; B.1-6.; C.; D.; E. 1.-3.**](https://optometriceducation.org/files/EducationalTechnologyGuidelines.pdf) [17]

To provide a foundation for life-long learning, the successful optometry school graduate should be able to do the following:

*A.* Define and articulate the need for information and the nature and extent of the information needed.

*B.* Access needed information effectively and efficiently.

1. Identify a variety of types of formats and potential sources of information. Select the most appropriate information resources and tools available, and demonstrate awareness of these resources, their content, and the information needs they can address.
2. Retrieve information online or in person using the most appropriate method and retrieval systems for accessing the needed information. Relevant bibliographic resources include MEDLINE (http://PubMed.gov/), VisionCite (VisionCite.com) and other databases, textbooks and reference sources. Other relevant resources include health sciences internet resources, optometry-based technology such as patient simulation software, diagnostic tools, electronic patient records, and digital imaging technology.
3. Construct and implement effectively designed search strategies. Perform database searches using logical (Boolean) operators in a manner that reflects understanding of methods of scientific inquiry, biomedical language, terminology, and the relationships among biomedical terms and concepts.
4. Reevaluate the nature and extent of the information need. Refine search strategies to improve relevance and completeness of retrieved items if necessary.
5. Extract, record, and manage the information and its sources. Use a standard bibliographic application to download citations from a search and organize them into a personal database.
6. Retrieve patient-specific information from a clinical information system. Demonstrate knowledge of available sources of decision support, ranging from textbooks to diagnostic systems to advisories issued by a computer-based patient record.

*C.* Evaluate information and its sources critically and incorporate selected information into his or her knowledge base and value system.

1. Filter, evaluate, and reconcile information, demonstrating the following:
   1. Knowledge of the factors that influence the accuracy and validity of information in general.
   2. The need to use multiple information sources for problem solving; the ability to discriminate and evaluate types of information sources in terms of their currency, format (e.g., review v. original article), authority, relevance, and availability.
   3. The ability to weigh conflicting information from several sources and reconcile the differences.
   4. The ability to critically review a published research report.
   5. The ability to synthesize main ideas to construct new concepts, determine the value added, contradictions, or other unique characteristics of the information.
   6. The ability to make decisions based on evidence, when such is available, rather than opinion.
   7. The ability to validate understanding & interpretation of the information through discourse with other individuals, subject area experts, and/or practitioners.

*D.* Use information effectively to accomplish a specific purpose.

1. Effectively employ written, electronic and oral communication, demonstrating the following:
   1. The ability to use software to create visual materials that effectively support oral presentations.
   2. The ability to create a handout that includes simple graphics and tables for use in teaching or patient information.

*E.*  Understand many of the economic, legal, and social issues surrounding the use of information and information technology; access and use information ethically and legally.

1. Demonstrate knowledge of copyright and intellectual property issues, especially with regard to materials that are retrieved electronically. Acknowledge the use of information sources in communicating the product or performance.
2. Follow laws, regulations, institutional policies, and etiquette related to the access and use of information resources and electronic communications.
3. Maintain a healthy skepticism about the quality and validity of all information. (This includes recognition that technology which provides new capabilities also has the potential to introduce new sources of error.)
